# Conditional transcriptional relationships may serve as cancer prognostic markers

**DOI:** 10.1186/s12920-021-00958-3

**Published:** 2021-12-02

**Authors:** Hui Yu, Limei Wang, Danqian Chen, Jin Li, Yan Guo

**Affiliations:** 1 Department of Internal Medicine, University of New Mexico, Albuquerque, NM 87131 USA; 2 Key Laboratory of Tropical Translational Medicine of Ministry of Education, Hainan Medical University, Kaikou, Hainan 571199 China; 3 College of Intelligent Systems Science and Engineering, Harbin Engineering University, Harbin, 150001 Heilongjiang China; 4 Key Laboratory of Resource Biology and Biotechnology in Western China, School of Life Sciences, Northwest University, Xi’an, 710069 Shaanxi China

**Keywords:** Cancer prognosis, Correlation by Individual Level Product, Conditional transcriptional relationships

## Abstract

**Background:**

While most differential coexpression (DC) methods are bound to quantify a single correlation value for a gene pair across multiple samples, a newly devised approach under the name Correlation by Individual Level Product (CILP) revolutionarily projects the summary correlation value to individual product correlation values for separate samples. CILP greatly widened DC analysis opportunities by allowing integration of non-compromised statistical methods.

**Methods:**

Here, we performed a study to verify our hypothesis that conditional relationships, i.e., gene pairs of remarkable differential coexpression, may be sought as quantitative prognostic markers for human cancers. Alongside the seeking of prognostic gene links in a pan-cancer setting, we also examined whether a trend of global expression correlation loss appeared in a wide panel of cancer types and revisited the controversial subject of mutual relationship between the DE approach and the DC approach.

**Results:**

By integrating CILP with classical univariate survival analysis, we identified up to 244 conditional gene links as potential prognostic markers in five cancer types. In particular, five prognostic gene links for kidney renal papillary cell carcinoma tended to condense around cancer gene *ESPL1*, and the transcriptional synchrony between *ESPL1* and *PTTG1* tended to be elevated in patients of adverse prognosis. In addition, we extended the observation of global trend of correlation loss in more than ten cancer types and empirically proved DC analysis results were independent of gene differential expression in five cancer types.

**Conclusions:**

Combining the power of CILP and the classical survival analysis, we successfully fetched conditional transcriptional relationships that conferred prognosis power for five cancer types. Despite a general trend of global correlation loss in tumor transcriptomes, most of these prognosis conditional links demonstrated stronger expression correlation in tumors, and their stronger coexpression was associated with poor survival.

**Supplementary Information:**

The online version contains supplementary material available at 10.1186/s12920-021-00958-3.

## Background

In more than a decade, a genre of bioinformatics approaches to transcriptional correlation changes has been steadily progressing. By focusing on the gene–gene relationships rather than individual genes in isolation, these algorithms/methods are collectively termed Differential Coexpression (DC) approaches, in contrast to the mainstream Differential Expression (DE) approaches. One of the most successful applications in this lineage is R package WGCNA [1], which has been utilized thousands of times after its release in 2008. Nevertheless, it is worth noting that WGCNA was not originally purported straightforward towards correlation changes; rather, WGCNA seeks strong correlations overarching heterogeneous experimental conditions with the additional requirement of cross-conditional gene differential expression. The vast majority of DC methods, mostly arriving later than WGCNA, are projected towards correlation changes directly, i.e., gene–gene correlations that change substantially between conditions/phenotypes. Such differential or dynamic transcriptional relationships were recently denoted as conditional relationships [2].

As summarized in several reviews [3–5], DC methods hold unprecedented promises for unravelling disease dysregulation mechanisms and prioritizing supplemental disease markers. At a very abstract level, DC methods can be classified by their primary analysis entities (genes, gene pairs, or gene sets), the delimitation of candidate entities (a priori defined or data-driven), and the number of experimental conditions that can be compared simultaneously (two or multiple) [2]. Regardless of all these classification angles, nearly all DC methods share an essential feature that they summarize one correlation index under one condition and then focus on the change of such a correlation index between conditions. Such foremost bind to condition-wise correlation values imposes unavoidable methodological limitations. For example, it is not recommended to exercise DC analysis when there are few samples per condition (say, n < 10), or when sample sizes are considerably imbalanced between conditions. Recently, a study of GTEx expression datasets denounced the independent value of DC approaches, declaring that most DC relationships can be more parsimoniously explained by DE of the involved genes [6]. On the other hand, though, consecutive benchmark studies are evaluating a growing body of DC methods and they generally approved of the independent or complementary benefits brought forth by DC methods [2, 7, 8].

In 2019, an innovative DC method was proposed under the name of “Correlation by Individual Level Product (CILP)” [9]. Traditionally, most DC methods rely on the Pearson Correlation Coefficient (PCC) to summarize the gene–gene correlation level for a condition that consists of multiple samples. Very creatively, Lea and colleagues sought to project the summary PCC value to individual post-scaling product values for separate samples in one condition. The CILP method is justified by an intrinsic mathematic property of PCC; PCC is equal to the average element-wise product of two traits measured across samples, after each trait is mean centered and scaled. Intuitively, the element-wise products of two genes’ post-scaling expression profiles appear as compelling sample-wise correlation measures between the two concerned genes. CILP thus reasonably defines sample-wise “product correlation” values for any pair of genes, effectively formulating a kind of “pair correlation matrix” that is analogous to a “gene expression matrix” in appearance. With this conceptual revolution, theoretically all statistical methods customized for DE analysis can be transferred to the DC framework without compromising their methodological beauty. For instance, Lea and colleagues demonstrated the successful implementation of linear regression model in the DC context with convenient incorporation of sample-wise clinical covariates [9].

Survival analysis correlates omics data with patient prognosis and holds promises for nominating prognosis markers. Survival analysis has been playing an essential and decisive role in bio-medical research [10, 11]. In the CILP introductory study [9], the authors identified metabolite pairs of strongest correlation loss and validated their prediction power for future development of metabolism disease (Fig. 3d in [9]). However, according to personal correspondences with Dr. Amanda Lea, the prognosis validation analysis was based upon levels of individual metabolites rather than the coexpression levels of metabolite pairs. Per Dr. Lea’s explanation, it would be more troublesome and costly to validate the coexpression of few metabolites in relation to the whole metabolome than to measure the levels of the few metabolites alone. However, given the fact that the few metabolites were prioritized in terms of DC analysis, their prognosis value would be much more logically and convincingly validated from the DC perspective than the DE perspective.

Motivated by the greatly widened opportunities offered by the novel CILP framework, and also intrigued by the logical defect that we appreciated in Dr. Lea’s survival analysis [9], we decided to integrate CILP with classical univariate survival analysis where the concerned entity is not a typical gene but a gene pair/link. We took advantage of the comprehensive transcriptome and survival data of hundreds of patients from The Cancer Genome Atlas (TCGA), which spans dozens of cancer types thus offering a pan-cancer analysis opportunity. In brief, this study was primarily aimed to explore whether CILP is capable of identifying conditional transcriptional relationships as cancer prognostic markers; meanwhile, we also revisited the controversial subject of mutual relationship between the DE approach and the DC approach.

Of note, cancers are commonly regarded as a kind of network-based diseases with synthetic lethal interactions holding promises for new therapeutic solutions [12]. Currently, the computational efforts directed towards synthetic lethal interactions mainly exploit double knockout libraries of yeast or the mutual exclusivity of somatic mutations revealed in thousands of human cancer genomes. We advocate CILP as a promising method to identify conditional gene links from data at the transcriptome level, and we see that CILP is technically applicable to data at the genomics level. It is our belief that a CILP-based strategy may also be exploited to help discover synthetic lethal interactions in cancer genomes.

## Methods

### Raw data and data preprocessing

We retrieved whole-transcriptome RNA-Seq data for multiple cancer types from TCGA via R package TCGA2STAT [13]. RNA-Seq data were log-transformed and quantile-normalized in a homogeneous sample group (e.g., tumor samples of one cancer type). Clinical data for the same patient cohorts were also obtained via TCGA2STAT. Thirteen cancer types (BRCA, COAD, COADREAD, HNSC, KICH, KIPAN, KIRC, KIRP, LIHC, LUAD, LUSC, PRAD, and THCA) were analyzed in parallel in this study, because they had at least 20 matched samples for the tumor and normal conditions. A full description of the 13 cancer types can be found in Table 1.

**Table 1:** Detailed information on cancer types involved in this study

Cancer type (abbreviated)	Cancer type (full name)	Sample size in paired comparison	Sample size in survival analysis	Percentage of censored samples (%)
BRCA	Breast invasive carcinoma	112	980	89.1
COAD	Colon adenocarcinoma	26	N/A*	N/A*
COADREAD^§^	Colorectal adenocarcinoma	32	N/A*	N/A*
HNSC	Head and neck squamous cell carcinoma	43	477	60.6
KICH	Kidney chromophobe	25	N/A*	N/A*
KIPAN^§^	Pan-kidney cohort (KICH + KIRC + KIRP)	129	759	74.8
KIRC	Kidney renal clear cell carcinoma	72	461	67.9
KIRP	Kidney renal papillary cell carcinoma	32	257	85.6
LIHC	Liver hepatocellular carcinoma	50	320	70.0
LUAD	Lung adenocarcinoma	58	449	65.0
LUSC	Lung squamous cell carcinoma	51	444	59.5
PRAD	Prostate adenocarcinoma	52	445	97.8
THCA	Thyroid carcinoma	59	442	97.3

^§^COADREAD and KIPAN were not among original TCGA panel of cancer types; they were complex cancer types derived by authors of R package TCGA2STAT

*Three cancer types did not return at least ten significant DCLs in their respective CILP analysis, so they were not screened for prognosis DCLs via survival analysis

From COXPRESdb (v7) [14], we downloaded the coexpression gene lists and “coexpression supportability” for all human genes. Coexpression supportability of a gene reflects the robustness of its coexpressed gene list across different microarray/RNA-Seq platforms, with vast cross-study samples taken into consideration [15]. COXPRESdb recognized around 7000 human genes as high-supportability genes (supportability = 3). The original COXPRESdb data were processed to yield 691,471 conserved gene coexpression pairs, which involved only the top 1% coexpressed partners of each high-supportability genes.

### To study global correlation change trend in tumors

For each cancer type, we calculated condition-wise PCC values for each of the 691,471 conserved gene pairs, where a “condition” denotes either the normal group or the tumor group. Therefore, for each cancer type, two series of paired PCC values were derived for all conserved gene pairs. We performed linear regression analysis between the paired PCCs, and plotted the regressed linear model to emphasize the qualitative property of slope (slope < 1 or not). Additionally, by examining the increase/decrease directionality of the absolute PCC values in the normal-to-tumor comparison, we labelled each conserved gene pair as either Strengthened or Weakened, and thus divided ~ 700 K conserved coexpression pairs into a Strengthened part and a Weakened part.

It was impractical to visually delineate 700 K gene pairs simultaneously; therefore, for the sake of meaningful visualization, we transiently switched to all possible gene pairs formed among the 500 most differentially expressed genes (DEGs). R package limma [16] was employed to identify the top 500 DEGs in a paired normal-versus-tumor setting, and PCCs for each of the 124,750 inter-DEG pairs were calculated. A cross-conditional asymmetrical expression correlation heatmap was plotted for each cancer type surrounding these 500 DEGs.

### To investigate reproducibility of correlation measures and differential coexpression results

To conduct the reproducibility analyses in BRCA, we first identified 2051 loosely defined DEGs (10% of the total number 19,790) in a paired tumor-versus-normal comparison (n = 112), using R package limma. Following a half-thresholding strategy [17], we narrowed down gene pairs to 39,038 tuples whose absolute PCC value in either condition was ranked within the highest 1% of all 2,102,275 pairs among the 2051 DEGs. For each gene pair, by comparing the signs of two PCC values derived from the 112 normal samples and the 112 paired tumor samples, respectively, we classified it as differently signed, same-signed positive, or same-signed negative. A single round of CILP analysis was performed to compare the 112 normal samples and the 112 paired tumor samples, where we fitted the product correlation values on the sample grouping variable and adjusted for the expression values of the two gene entities. Differentially Coxpressed Links (DCLs) were identified as those showing False Discovery Rate (FDR) < 0.1 as converted from the original CILP p-value with the Benjamin-Hochberg method. Furthermore, these DCLs were separated into DE-dependent DCLs and DE-independent DCLs, where the former showed significant dependence of product correlations on at least one gene entity (FDR < 0.1). To conduct DC analysis repeatedly for reproducibility assessment, we split the whole 1093 BRCA tumor samples into ten folds of roughly equal sizes (n = 112 for nine folds and n = 85 for one fold), and matched each fold of tumor samples with the same set of normal samples (n = 112) and performed ten rounds of CILP analysis in a non-paired comparison setting. Gene pairs with CILP-derived FDR < 0.1 were deemed as DCLs, and we counted the DCL recurrence (times of showing DC significance) across the ten repeated analyses.

### To identify conditional links in normal-to-tumor transition and further discriminate prognosis conditional links

For each of the 13 cancer types which sufficed the sample size requirement (n ≥ 20), we performed CILP analysis of each conserved coexpression gene pair to compare the product correlation values between the paired tumor and normal samples. The p-values out of the linear regression model (which degenerated to paired t-test in this case) were adjusted to FDR, and FDR ≤ 0.3 was imposed to accredit DCLs, or conditional links.

For ten cancer types, we examined the prognosis value of each DCL. Subject identities of the tumor RNA-Seq data and those of the survival data were matched, and the subjects who contributed paired samples in the DCL identification analysis above were excluded. Across the remaining tumor samples with concurrent RNA-Seq data and survival data, we centered and scaled the log-scale expression level and derived product correlation values for each DCL. The product correlation values were dichotomized to high-coexpression and low-coexpression by the across-cohort median value, and the binary coexpression values were correlated with overall survival data through the log-rank test. The p-values out of survival analysis were adjusted to FDR; again, FDR ≤ 0.3 was imposed to ascertain prognosis DCLs, i.e., conditional links that confer survival predictability.

We denoted the genes involved in DCLs as Differentially Coexpressed Genes (DCGs). We wanted to check if DCGs tended to be more differentially expressed across the whole spectrum of genes; in other words, we would like to see if there was a correlation between the differential coexpression and the differential expression of genes. We first applied limma on all 7 K high-supportability genes and obtained the differential expression *p*-values for each gene. The transformed differential expression *p*-values were treated as the RNK file in a Gene Set Enrichment Analysis (GSEA) [18], where we supplied a set of DCGs as the gene set of interest. GSEA thus returned us a *p*-value indicating the chance of the DCGs being randomly placed in the sorted gene panel. If the GSEA *p*-value was sufficiently small (say, *p* < 0.01), it would suggest a significant correlation between the differential coexpression and the differential expression of genes. GSEA was accessed as an R package (https://github.com/GSEA-MSigDB/GSEA_R).

## Results

### Overall decoherence in tumor transcriptomes is observed in BRCA and all other TCGA cancer types

In the CILP introductory study of metabolism diseases [9] and our study of chronic kidney disease [19], a global correlation loss trend was observed from a normal transcriptome to a diseased transcriptome. Coincidentally, years ago, the same trend was reported for five cancer types in an analysis of several GEO microarray datasets [20]. Here, we took the opportunity to verify/extend the possibly universal phenomenon of pathological co-transcription attenuation in a wider range of cancer types, using RNA-Seq data from TCGA.

**Fig. 1:**
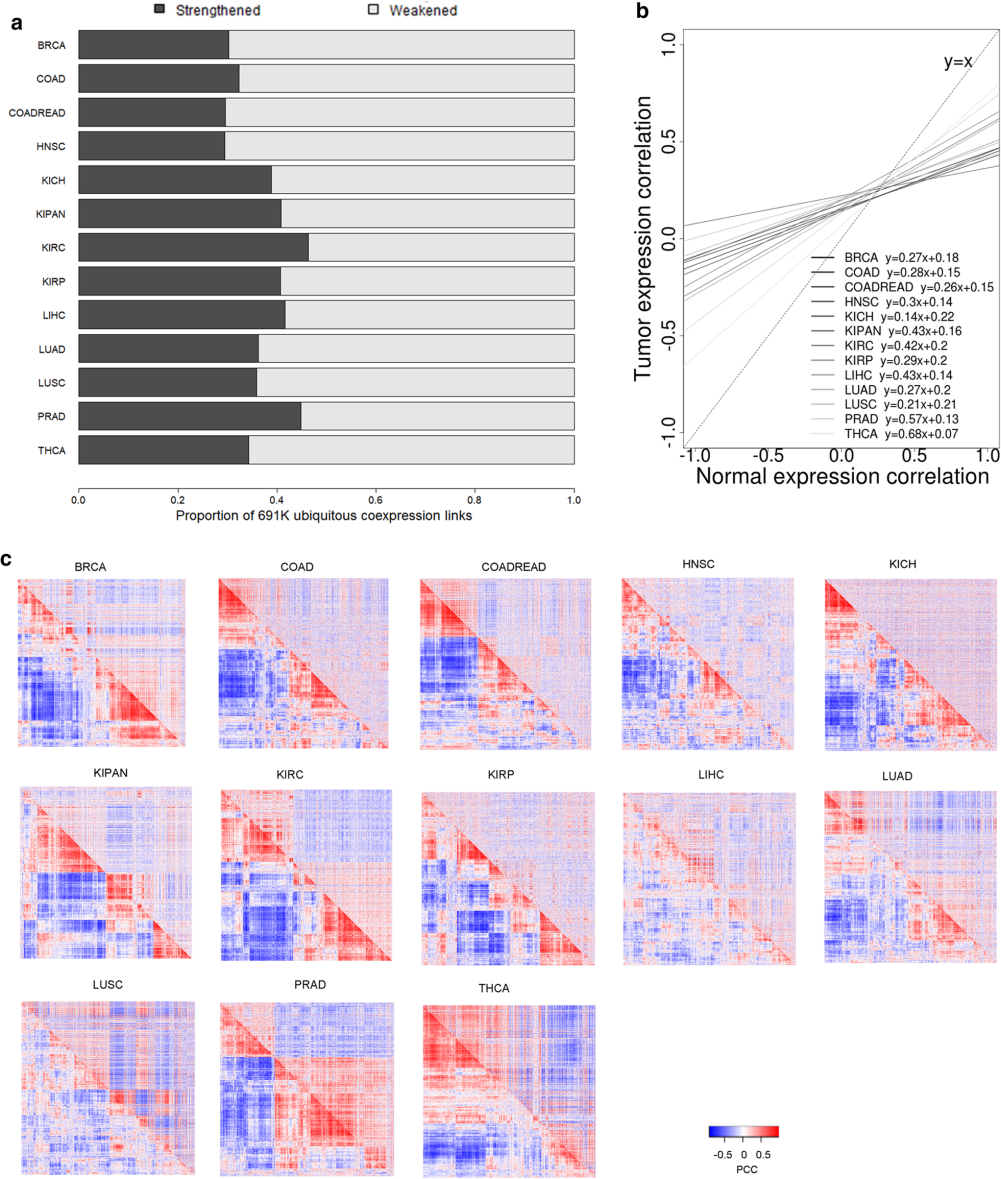
Global correlation losses dominated tumor transcriptomes in comparison with paired normal transcriptomes. **a** By considering the directionality of correlation change from normal to tumor, 691 thousand ubiquitous coexpression pairs were divided into a strengthened part and a weakened part. **b** Representations of linear regression models between normal PCC and tumor PCC. **c** Cross-conditional asymmetrical expression correlation heatmaps for 13 cancer types. PCC values for all possible gene–gene pairs formed among the top 500 differentially expressed genes were indicated for the normal phenotype (lower-triangle) and the tumor phenotype (upper-triangle), respectively. The order of genes in the rows was the same as the order in the columns, so that the spots symmetrically positioned off the diagonal line depicted the same pair of genes with possibly varied PCC values across phenotypes

To our expectation, we observed a predominant co-transcription attenuation in tumor as compared to paired normal, for 13 technically defined cancer types (Fig. 1). When we classified each of the ~ 700 K conserved coexpression gene pairs to either a Strengthened part or a Weakened part, all 13 cancer types received more than 50% Weakened gene pairs (Fig. 1a). When we visualized the linear regression model between paired PCCs from normal to tumor, all 13 cancer types demonstrated a slope less than 1 (Fig. 1b), certifying the same trend of dominant co-transcription attenuation. Lastly, when we visualized the paired PCCs for 500 DEGs in an asymmetric correlation heatmap that overarched the normal and tumor conditions, the global correlation loss trend was unambiguously revealed in a majority of the 13 cancer types (Fig. 1c). According to visual judgement, there was substantial correlation loss in eight cancer types (BRCA, COAD, COADREAD, HNSC, KICH, KIPAN, KIRC, KIRP), weak yet discernable correlation loss in two cancer types (LUAD and PRAD), and an ambiguous trend in two cancer types (LIHC and THCA). Taken together, these results from diverse angles or gene scopes suggested a possibly universal phenomenon of pathological co-transcription attenuation in a wide range of cancer types. This plausible global trend of pathological co-transcription attenuation is in concert with prior sporadic reports [9, 20, 21].

### Reproducibility of correlations and DCLs

Obviously, the success of a DC analysis is contingent upon robust coexpression quantification against variant same-natured sample sets. PCC is the most commonly practiced condition-wise correlation metric, but it is susceptible to noise samples [22]. A recent study particularly addressed the problem of variable PCCs resulting from different sample subsets [23]. The BRCA RNA-Seq dataset has a unique, extremely imbalanced sample structure in terms of tumor versus normal ratio (1093 vs. 112). We used to take advantage of this unusual sample structure to study various classifiers’ tolerance to increasing severity of class imbalance [24]. Now we leveraged the 10:1 sample size ratio to assess the reproducibility of correlation measures (PCC) and DCL results across ten repetitive datasets which each used a different fold of tumor samples.

Firstly, we observed high and stable correlation of gene pair PCCs across the ten folds of BRCA tumor samples (Additional file 1: Fig. S1): the cross-fold PCC of gene pair PCCs ranges in 0.76–0.86. The high concordance of gene pair PCC values between alternative tumor subsets endorsed the sample homogeneity within the same condition (tumor or normal) and consistency in RNA-Seq data preprocessing. More importantly, the reproducible correlation values established the foremost validity of applying a DC approach to identify meaningful DCLs.

**Fig. 2:**
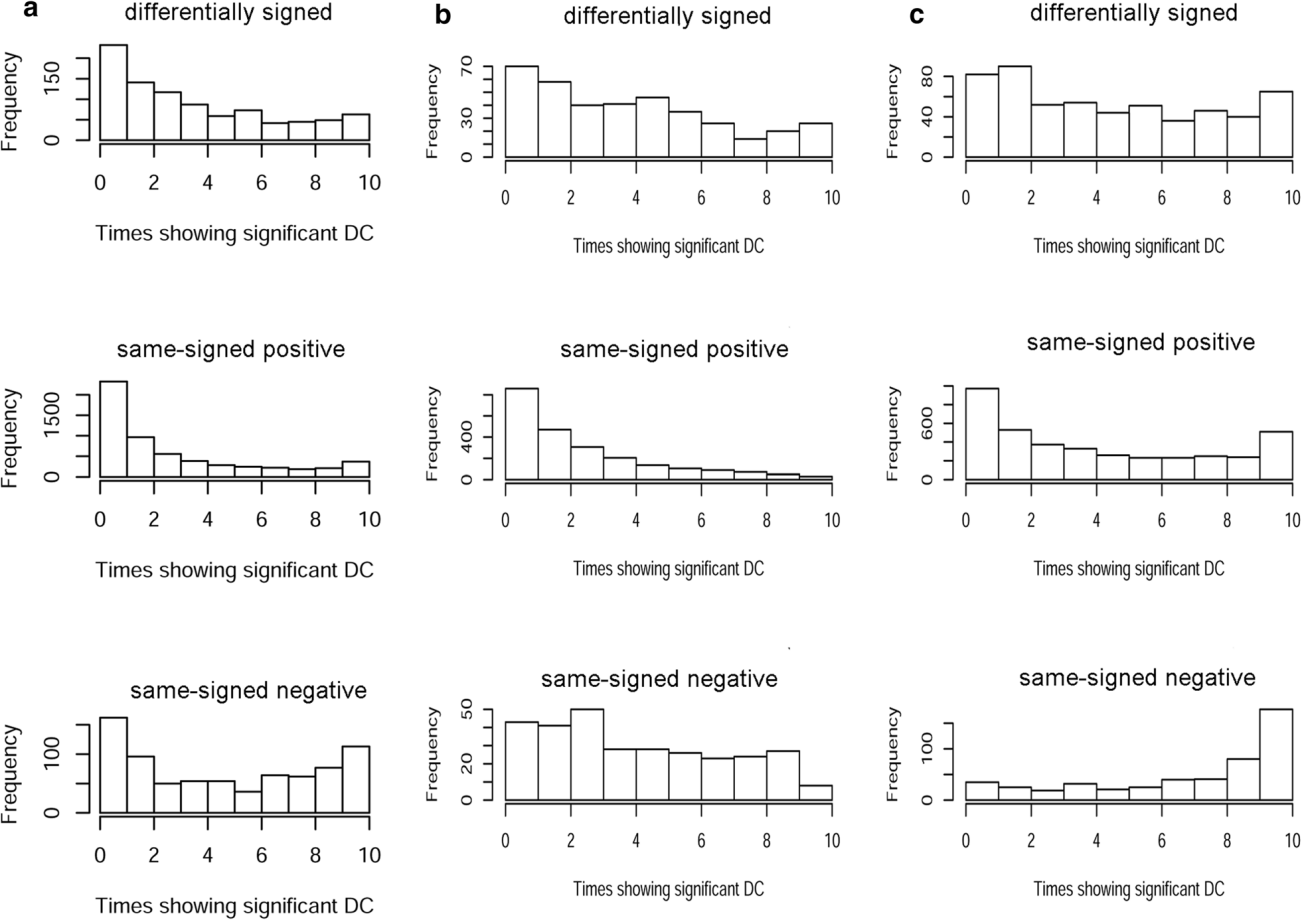
Distribution of DCL recurrence across ten scenarios of BRCA versus normal comparisons, where each time a different fold (1/10) of whole tumor samples were recruited. **a** All reference DCLs identified from the paired-comparison setting, which were further divided into three subsets according to the paired correlation signs. **b** The DE-independent component of reference DCLs. **c** The DE-dependent component of reference DCLs

Next, we performed CILP analysis in ten runs where each time we matched a different fold of tumor samples (n = 112 or 85) with the same set of normal samples (n = 112). Before the ten rounds of repetitive CILP analysis, we performed one round of CILP analysis on the paired tumor-versus-normal dataset, where two variables for the gene expression values of the two gene entities were incorporated alongside the sample grouping variable. This sample-paired CILP analysis was exerted for obtaining a set of reference DCLs that were separated into a DE-dependent subset and a DE-independent subset. Next, CILP analysis was repeatedly performed on ten rounds of non-paired tumor-versus-normal datasets, and we summarized the recurrence statistics for each reference DCL based on their occurrence as DCLs in the ten result sets. DCL recurrence takes value from [1, 10], the higher the more reproducible. We plotted the distribution of DCL recurrence for three components of reference DCLs, classified by the directionality of correlation signs (Fig. 2). Unsurprisingly, in many dissections of the data, a reference DCL was most likely to appear in only one of the repetitive analysis results. This general trend was refuted in DE-dependent, negatively correlated DCLs (Fig. 2c), where most reference DCLs tended to be confirmed in all ten non-paired CILP analyses. This led us to suppose that, in general, differential negative correlations could be more reproducible than differential positive correlations.

From all 39,038 half-thresholded coexpression links, the sample-paired CILP analysis identified 8397 DE-dependent DCLs and 5859 DE-independent DCLs. While DE-dependent DCLs outnumbered DE-independent DCLs, their quantity advantage is weak, at a percentage of 59%, noticeably lower than the previously alleged 75% [6]. According to the distribution patterns of DCL recurrence, we seemingly verified the prior observation [6] that DE-dependent DCLs are more reproducible than DE-independent DCLs, especially for the most noteworthy subset of negatively correlated DCLs (Fig. 2b vs. Figure 2c). However, we designed another way to study the relationship between DEGs and DCGs, finding no significant evidence of mutual correlation (see next section).

### Conditional relationships emerging as cancer prognostic markers

For each of the 691,471 conserved coexpression pairs (derived from COXPRESdb [14]; see Methods), we performed CILP analysis to identify differentially coexpressed links (DCLs) with FDR < 0.3. Of all 13 analyzed cancer types (Table 1), ten cancer types returned at least ten DCLs each (Table 2). Details of the DCLs for five cancer types (HNSC, KIPAN, KIRC, KIRP, and THCA) were provided in Additional file 2: Table S1.

**Table 2:** Differentially coexpressed links (DCLs), differentially coexpressed genes (DCGs), and correlation between DCGs and differentially expressed genes (DEGs)

	DC analysis results				Survival analysis results			
	#DCL	#DCG	Maximum *p*-value of DCLs	GSEA *p*-value*	#DCL	#DCG	Maximum *p*-value of DCLs	GSEA *p*-value*
BRCA	4920	2538	5.3e − 7	N/A	0	0	N/A	N/A
HNSC	13	23	5.2e − 7	0.257	1	2	0.019	0.380
KIPAN	11,549	3950	5.4e − 7	N/A	244	331	2.6e − 5	0.210
KIRC	1213	971	5.3e − 7	N/A	7	11	0.0002	0.436
KIRP	21	34	5.1e − 7	0.930	5	8	0.010	0.071
LIHC	19	29	5.2e − 7	0.070	0	0	N/A	N/A
LUAD	134	173	5.3e − 7	0.823	0	0	N/A	N/A
LUSC	66	85	4.9e − 7	0.227	0	0	N/A	N/A
PRAD	16	27	5.1e − 7	0.303	0	0	N/A	N/A
THCA	13	21	4.9e − 7	0.856	1	2	0.015	0.614

Survival analysis results (DCGs and DCLs) were subsets of DC analysis results that met the criterion of FDR ≤ 0.3 in DCL survival analysis

*Only the cancer types that retrieved at least two DCGs were fed to GSEA analysis, and when the gene number was more than 500 we did not perform a GSEA analysis

**Fig. 3:**
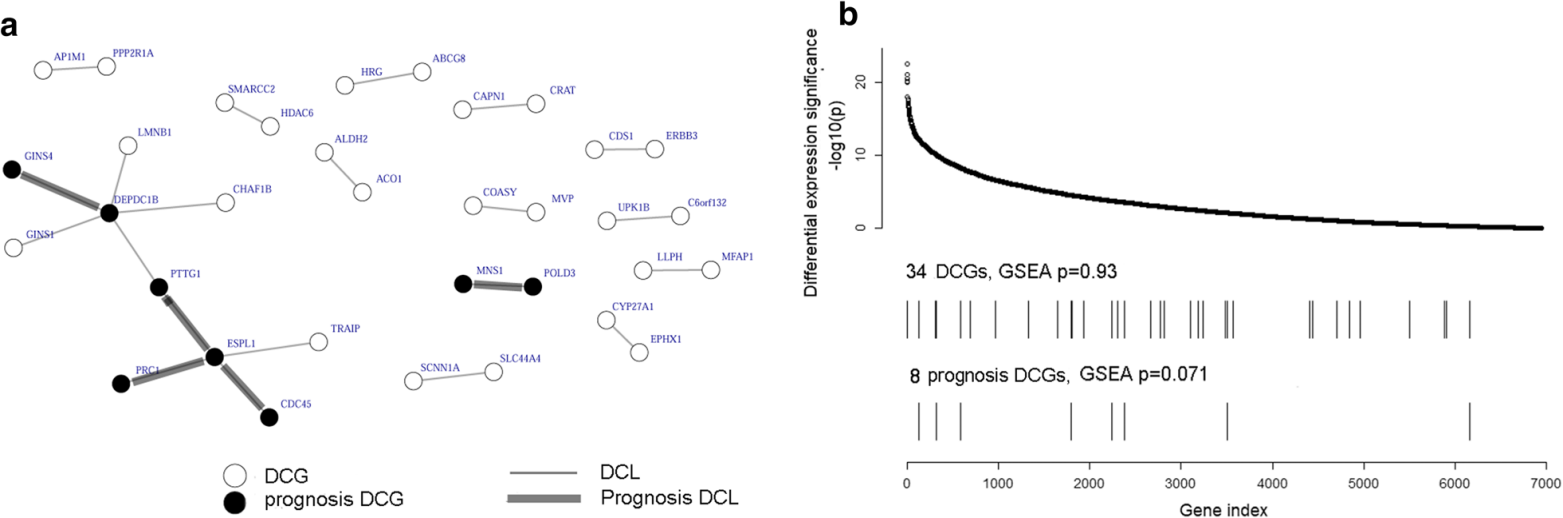
KIRP conditional links characterized with normal-versus-tumor differential coexpression and prognosis conditional links associated with overall survival. **a** Network of 21 DCLs (conditional links) with the 5 prognosis DCLs highlighted (thick edge and solid vertex) for association with survival. **b** Placement of DCGs and prognosis DCGs in the spectrum of ~ 7 K genes of decreasing differential expression significance. DCGs and prognosis DCGs corresponded to DCLs and prognosis DCLs in **a**, respectively. The correlation between DCGs/prognosis DCGs and gene differential expression significance was analyzed with Gene Set Enrichment Analysis (GSEA)

**Fig. 4:**
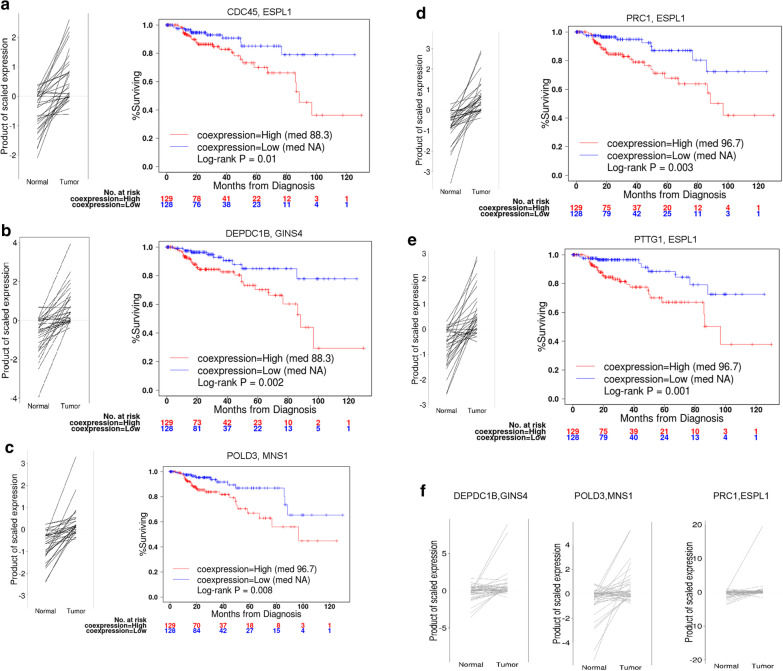
Five KIRP conditional links that were found with prognosis predictability. **a**–**e** Differential coexpression trend and survival discrimination of the five conditional links. The left panel shows how the product correlation values change for each paired subject from the normal sample to the tumor sample; the right panel shows the survival difference between two sub-cohorts of cancer patients separated by the median product correlation value of the same gene pair. **f** Three of the five conditional links showed statistically significant (paired t-test, *p* < 0.05) and same-direction coexpression changes in renal cell cancer RNA-Seq data from International Cancer Genome Consortium

For each DCL of each cancer type, we performed survival analysis on tumor samples with the normal-paired subjects excluded. The log-rank test p-value was adjusted to FDR and FDR < 0.3 was required to call prognosis DCL. At this stage, five cancer types returned 1–244 prognosis DCLs, whereas the other five cancer types returned null sets (Table 2). The prognosis DCLs tended to inter-connect to limited number of genes, as exemplified in KIRP where five DCLs forming three discrete network modules (Fig. 3a). We performed a small-scaled validation experiment on the DC of 12 prognosis DCLs associated with KIRC and KIRP using the RNA-Seq data of renal cell cancer (RECA-EU) from International Cancer Genome Consortium (https://dcc.icgc.org/projects/RECA-EU). Using the CILP method, we found eight out of the total 12 prognosis links of KIRC and KIRP showed statistically significant elevated correlation from normal to tumor (two-sided paired t-test, *p* < 0.05), which included three of the five prognosis links for KIRP (Fig. 4f).

All five prognosis DCLs for KIRP showed increased coexpression in tumor versus normal, and concordantly, patients with high coexpression of these gene pairs had significantly shorter overall survival (Fig. 4). The STRING database [25] confirmed functional inter-connection for three out of the five prognosis gene pairs, namely *CDC45:ESPL1*, *ESPL1:PTTG1*, and *ESPL1:PRC1*. These three links formed a compact network module (Fig. 3a), with *ESPL1* emerging as a hub. The COSMIC database indicates that somatic mutations in the *ESPL1* gene was related to human lung and kidney cancers [26]. One partner gene of *ESPL1, PTTG1*, was proved to be an oncogene in renal cell carcinoma [27, 28] and other cancer types [29–31].

For each cancer type, from the DCL superset and the prognosis DCL subset, we derived the so-called DCGs and prognosis DCGs, as those genes incident to the DCLs (Table 2). We employed the well-known GSEA algorithm to test if DCGs tended to demonstrate more remarkable DE. For the three cancer types (HNSC, KIRP, and THCA) which returned moderate number of DCGs, no significant correlation between gene differential coexpression and gene differential expression was observed (minimum *p* = 0.257, Table 2). Speaking of the prognosis DCGs, all five GSEA-applicable cancer types returned non-significant *p* values (minimum *p* = 0.071, Table 2, Fig. 3b). In summary, in our empirical investigation of five cancer types, genes’ differential coexpression attribute is not dependent on their differential expression attribute. Here, we arrived at a conclusion that is seemingly contradictory to what was declared by the related study [6] and verified by us in the previous Results section.

## Discussion

In this pan-cancer differential coexpression analysis, we first revisited the presumably general phenomenon of coexpression loss in tumors. In light of ~ 700 K conserved gene pairs surrounding highly supported genes curated by COXPRESdb, we found that each of the 13 surveyed cancer types showed a less-than-one slope for their linear models of tumor PCCs on normal PCCs. This meant that the assertion of tumor correlation attenuation may be extended from 5 [20] or 3 [32] to at least 11 cancer types (Table 1, excluding the two complex cancer types). To increase confidence in this extended assertion, we also examined and confirmed the same trend with respect to the all-possible gene pairs among the top-500 DEGs. Overall, we feel confident to propose the extended assertion that a global correlation loss is present in tumor transcriptomes for more than ten cancer types. Very recently, nasopharyngeal carcinoma [33], an under-studied cancer type, has also been found with overwhelming transcriptional correlation losses. A possible explanation of such a pan-cancer global correlation attenuation may be the increased heterogeneity in tumor tissues.

Of note, the pan-cancer global correlation attenuation took place in both positive correlations and negative correlations—i.e., the magnitude of absolute correlation values was generally reduced in tumor samples. At times, negative expression correlations were overlooked or downweighted [21]. Even in the CILP introductory study, the correlation lessening trend in negative correlations was not explicitly underscored. Reassuringly, a study of connectivity loss in three cancer types gave special attention to negative correlations and revealed a same trend of connectivity loss for negative correlations [32]. In our cross-fold BRCA study, we even found that negatively-correlated DCLs were more reproducible than the positively-correlated DCL counterparts. While we did not trace further with the subset of negatively-correlated DCLs herein, future works may follow up to investigate possible coherent functions represented by negative DCLs, such as extracellular space related methylation targets in colorectal carcinoma as proposed in the related study [32].

We tried to revisit the controversial topic of mutual relationship between DE results and DC results. The DE analysis has always been the mainstream and foremost solution to a transcriptome study; the DC approach is typically touted as a beneficial complement to the default DE resolution. The non-trivial, added value offered by a DC analysis has been repeatedly discussed in early studies [34–37]. Our own study of Type-II-diabetes microarray data even found a DC analysis outperformed the traditional DE analysis to enrich drug targets [17]. In the present study, we empirically demonstrated in five cancer types that the genes involved in prognosis-significant conditional relationships were not conspicuous in terms of their differential expression attribute. This observation added to the cumulative evidence that a DC analysis can uncover additional biological insights that might be otherwise missed by a traditional DE analysis. Although we reproduced the same phenomenon that a majority of conditional relationships are confounded with or explainable by (differential) expression of the gene entities [6], this confounding/correlation between DC and DE does not mean the genes involved in the conditional relationships can simply by retrieved through a traditional DE analysis. Actually, according to the definition of the product correlations by CILP, it is unsurprising that the sample-wise product correlations may be significantly dependent on the expression vector of either gene from the pair. Dependence of the product correlation on the gene expression does not necessarily translates to dependence of the gene expression on the sample grouping, which explains why DE-dependent conditional relationships still capture genes of survival predictability which did not stand out in a DE analysis.

Most importantly, we demonstrated the integration of the novel CILP method with the classical survival analysis in 13 cancer types, and successfully fetched conditional transcriptional relationships that conferred prognosis power for five cancer types. We had designed this study as a preliminary proof-of-concept trial, with the primary goal of confirming our hypothesis that conditional relationships, i.e., gene pairs of remarkable transcriptional rewiring, may be sought as quantitative prognostic markers for human cancers. At a rather permissive FDR threshold of 0.3, tens to thousands of conditional links survived CILP analysis, and after the next survival analysis step, 1–244 conditional links survived the same permissive FDR threshold of 0.3. Due to our pan-cancer analysis scope, we did not fine-tune the parameters involved in diverse analysis steps, or tweak the threshold values for focused cancer types. In fact, the FDR of the five prognostic DCLs retrieved for KIRP was 0.17, much lower than the threshold of 0.3. Future works targeting a specific cancer type may need to configure and optimize the threshold values, the gene scope, and the possible pre-filtration of gene pairs. The “pair correlation matrix” conceptualized by CILP also enables convenient incorporation of various sample-wise co-variates, such as age and gender. For simplicity, we did not make this attempt in this study, but it is quite worthwhile to try incorporating such clinical variables, as well as certain theme-relevant variables (such as smoking status in lung cancer and sunburn in skin cancer), into CILP-supported DC studies.

We identified five conditional gene relationships for kidney renal papillary cell carcinoma. It appears as an interesting phenomenon that, despite a general trend of global correlation loss in tumor transcriptomes, all these five prognosis conditional links demonstrated stronger expression correlation in tumors and their stronger coexpression was associated with poor survival (Fig. 4). The five prognostic DCLs tended to condense around the well-known cancer gene *ESPL1*, and the transcriptional synchrony between *ESPL1* and *PTTG1* (another well-known cancer gene) tended to be elevated in patients of adverse prognosis. This particular gene pair, alongside other gene pairs identified in this study, awaits future validations with independent datasets. Future studies should also try to decipher the functional mechanisms underlying these prognosis-significant conditional transcriptional relationships.

## Conclusions

In this work, we integrated CILP with classical univariate survival analysis where the concerned entity is not a typical gene but a gene pair/link, and thereby successfully identified up to 244 conditional transcriptional relationships that conferred prognosis power for five cancer types. Despite a general trend of global correlation loss in tumor transcriptomes, we observed that most of these prognosis conditional links demonstrated stronger expression correlation in tumors, and that their stronger coexpression was associated with poor survival. In addition, we extended the observation of global trend of correlation loss in more than ten cancer types and empirically proved DC analysis results were independent of gene differential expression in five cancer types.

## Supplementary Information

**Additional file 1: Fig. S1.** Robustness of PCC values across ten folds of BRCA tumors.

**Additional file 2: Table S1.** Prognosis DCLs in five cancer types.

## Data Availability

The data that support the findings of this study are available from public repositories TCGA (https://portal.gdc.cancer.gov/) and ICGC (https://dcc.icgc.org/). Majority of the data analyses were performed using R × 64 3.4.2. All R code written for this manuscript is available from the corresponding author upon request.

## Abbreviations

CILP: Correlation by Individual Level Product
DC: Differential Coexpression
DCG: Differentially Coexpressed Genes
DCL: Differentially Coxpressed Links
DE: Differential Expression
DEG: Differentially Expressed Gene
FDR: False Discovery Rate
PCC: Pearson Correlation Coefficient
TCGA: The Cancer Genome Atlas
