# Supplementary figures and images for: Conditional transcriptional relationships may serve as cancer prognostic markers
Source: BMC Med Genomics. 2021 Dec 2;14(Suppl 2):101. doi: 10.1186/s12920-021-00958-3 (PMC8638091; doi:10.1186/s12920-021-00958-3)

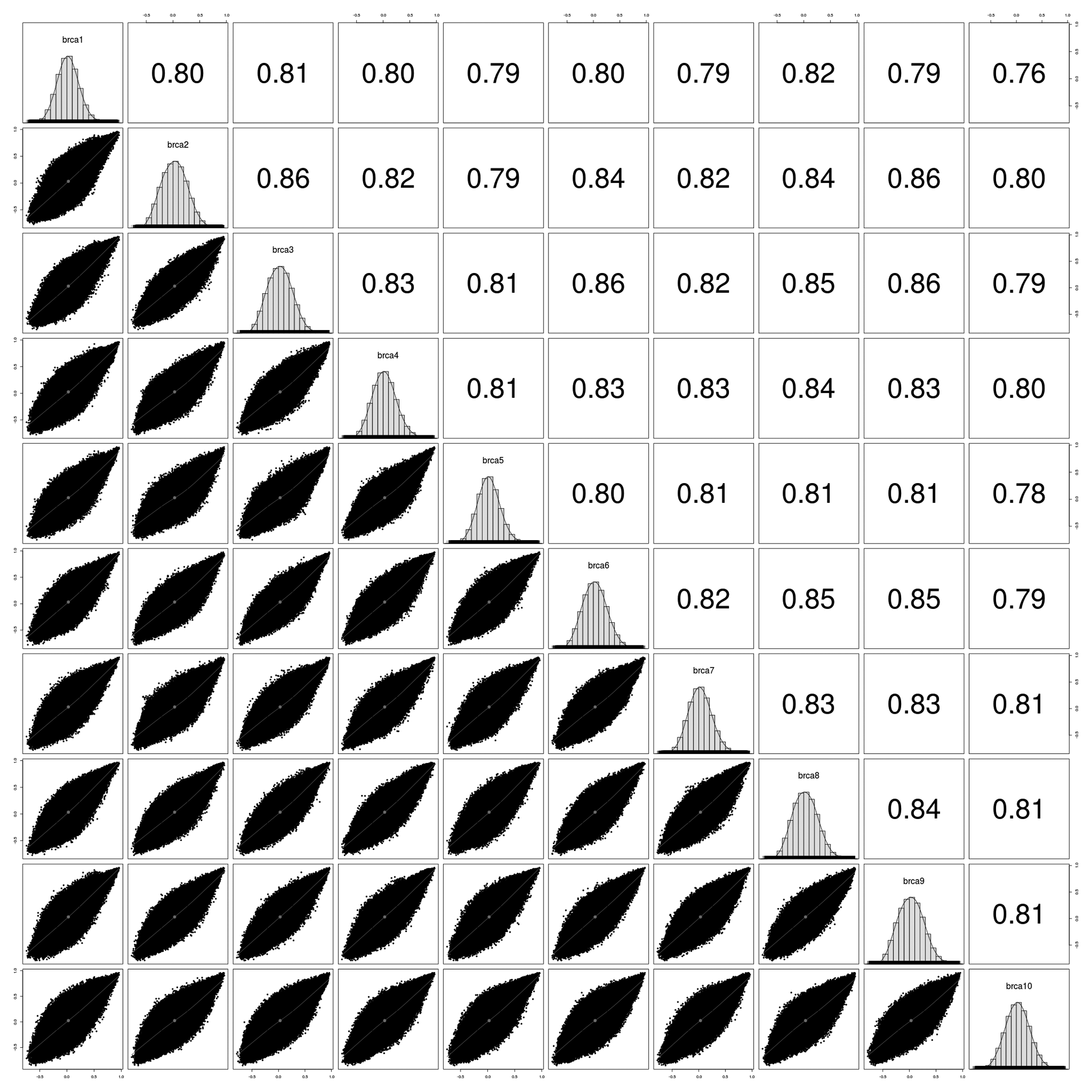

Supplement: Supplementary file 1 — Additional file 1: Fig. S1. Robustness of PCC values across ten folds of BRCA tumors. [file 12920_2021_958_MOESM1_ESM.tif]
